# Octopus: A low-cost, modular environmental sensing platform for makers

**DOI:** 10.1016/j.ohx.2026.e00760

**Published:** 2026-03-16

**Authors:** Oluwatobi Oyinlola, Åse Håtveit, Chada Elalami, Simone Mora, Ravish Dubey, Fábio Duarte, Jessica Biagioli, Matteo Gregis, Carlo Ratti

**Affiliations:** a Senseable City Lab, Massachusetts Institute of Technology, Cambridge, MA, USA; b Department of Computer Science, Norwegian University of Science and Technology, Trondheim, Norway; c ABC Department, Politecnico di Milano, Milan, Italy; d FAE Technology S.p.A., Bergamo, Italy

**Keywords:** Internet of things, Arduino, Low-cost sensors, Environmental sensors

## Abstract

Environmental monitoring is increasingly critical in urban and ecological contexts, yet existing tools are often costly or closed-source. Octopus is a full y open-source hardware platform designed to enable low-cost, modular environmental sensing for makers, educators, and citizen scientists. The system is built around a compact, 4-layer custom-designed PCB and a 3D printed enclosure, supporting Arduino-compatible microcontrollers (Nano 33 BLE Sense or Nicla Vision) and varied plug-and-play sensors, including particulate matter, GPS, temperature, and humidity. Octopus emphasizes ease of assembly, flexibility of deployment, and reproducibility. All hardware, firmware, and mechanical design files freely available. Devices can be built for less than 100 USD using widely available tools, significantly lowering the barrier to entry for environmental sensing. We provide complete documentation, including build instructions, firmware libraries, and example applications. Compared to existing citizen-science devices, Octopus offers an extensible and adaptable platform to support hands-on environmental research, educational use, and grassroots sensing initiatives.

## Specifications table

**Table:** 

Hardware name	Octopus

Subject area	• Environmental, educational tools

Hardware type	• Environmental Sensor • Computer science

Closest commercial analog	AirBeam2 (HabitatMap) and Clarity Node-S are commercially available environmental monitoring devices that offer similar sensing capabilities but are significantly more expensive and closed-source.

Open source license	Creative Commons Attribution 4.0 International Public License

Cost of hardware	Static Standard Use Case: *59.26 USD* Mobile Standard Use Case: *81.28 USD* Extended Use Case: *127.91 USD* Vision Use Case: *149.26 USD*

Source file repository	https://doi.org/10.17632/jxjfd7k8z4.2

OSHWA certification UID	US002770

	

## 1 Hardware in context

**Fig. 1:**
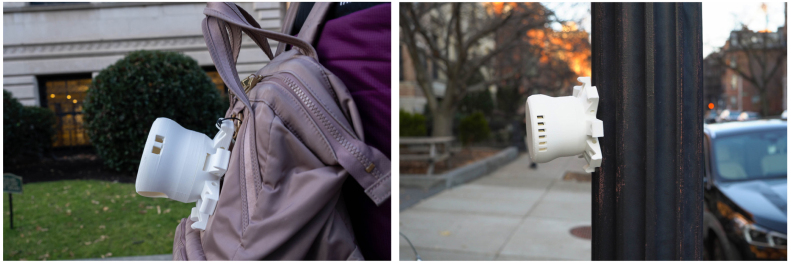
Octopus as a wearable (left) or mounted to a pole using a magnetic attachment (right).

With increasing interest in urban and ecological monitoring, there is a growing need for small, deployable, and inexpensive sensor devices. Many existing systems target specific domains (e.g. acoustic monitors, air-quality kits) or can be costly and proprietary [1]. Octopus offers a general-purpose environmental sensing platform: it combines low-cost construction with a highly modular design, targeting users such as do-it-yourself (DIY) enthusiasts, researchers, students, and hobbyists with basic technical skills. Its core design emphasizes easy fabrication and broad deployability: users can easily build the casing and electronics, add sensors as needed, and mount the device in a stationary or mobile contexts, in public and private spaces. Through the inclusion of a base with multiple bindings, including a standard 1/4”-20 UNC socket, the device is portable and can also be attached to different carriers (backpacks, bicycles, poles, windows) using wires, cords, suction cups, or magnets (see Fig. 1). This flexibility in binding to different surfaces and objects is what inspired the name “Octopus”, reflecting the animal’s ability to cling onto different surfaces with ease.

A “maker” can be described as an individual, from professional engineers and educators to students and hobbyists, who use emerging technologies and collaborative practices to create, modify, and share new products and solutions [2]. The Octopus follows a maker-oriented design approach, allowing it to be adapted for a variety of applications, including air pollution, microclimate sensing, and visual monitoring, without the need to re-implement the core hardware. A custom oval-shaped printed circuit board (PCB), combined with Arduino-standard pin-outs and modular connectors for various sensor modules, provide a versatile platform that supports a broad range of sensing and monitoring tasks (Fig. 10. This enables makers and DIY communities to concentrate on customizing sensors and applications, rather than having to redesign the underlying electronics for each new project. By engaging with the maker community, projects like Octopus not only accelerate the adoption of new technologies, but also foster a culture of open collaboration, democratizing access to environmental monitoring tools and knowledge.

### 1.1 Motivation and purpose

Similar products on the market have the disadvantage of providing only a limited number of data types, such as air temperature and humidity [3], or only chemical composition measurements [4]. The Smart Citizen System [5] was developed to create a fully open and flexible solution for environmental sensing, but it is limited by the addition of third-party components requiring modifications to the firmware and C++ programming expertise.

The primary contribution of Octopus lies in integration, accessibility, and documentation, rather than novel sensing methodologies. Our contribution focuses on lowering the barrier to entry for environmental sensing through hardware integration, and creating a platform that reduces setup time and learning curve, which is part of the key factors for a successful open hardware adoption [6]. Octopus reduces these barriers through several mechanisms: assembly time is about 30 min with basic electroncis tools; no soldering of sensor modules is required; pre-configured firmware examples in the Octopus Arduino library allow immediate data collection; Jupyter notebook for data analysis are provided; and the total cost under 100 USD (for the base configuration) is significantly lower than commercial alternatives like AirBeam2 or Clarity Node-S. In addition, the success of an adopted hardware platform depends on how it enables makers to build artifacts that were difficult to build before its introduction [6], [7]. Unlike most alternatives, Octopus supports both the Arduino Nano 33 BLE Sense [8] and Nicla Vision boards [9], enabling immediate data collection as soon as either board is connected. No additional breakout boards are required since the custom PCB provides all necessary connections. Comprehensive documentation and use-case guides are available for all skill levels. By leveraging off-the-shelf components and open-source design, Octopus makes environmental sensing available for educational and scientific purposes, enabling widespread participation and innovation. Octopus introduces plug-and play modular architecture that utilizes Qwiic-compatible connectors and enables sensor reconfiguration without soldering or rewiring. The Octopus architecture enables several use cases that are complex with existing platforms: (1) rapid deployment of mobile sensing networks with diverse mounting options (backpacks, bicycles, poles, windows or magnets) and support for both stationary and mobile sensor deployment; (2) vision-based environmental monitoring through the Nicla Vision support, enabling applications like visual AI models for image detection and classification; (3) multi-sensor correlation studies where parameters like temperature, humidity, particulate matter (PM), and GPS data can be collected simultaneously with synchronized timestamps; (4) educational settings where the same base hardware supports progressively complex projects.

### 1.2 Use cases

**Fig. 2:**
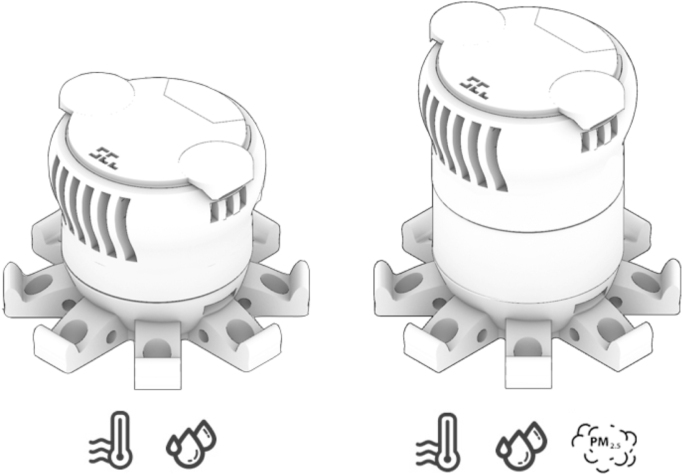
The two Octopus use cases: Standard (left) and Extended (right).

Octopus is designed to be modular and adaptable, allowing users to tailor the device to specific monitoring needs (see Fig. 2). To support different application scenarios, we define four primary use cases:

• **Static Standard Use Case (1)**: This entry-level setup includes the core PCB, an Arduino Nano 33 BLE Sense [8], and a rechargeable Li-ion battery. It is intended for stationary deployments where location tracking is not required, such as fixed-point temperature and humidity tracking. An accompanying Jupyter notebook for data analysis is available in the source file repository.
• **Mobile Standard Use Case (2)**: This setup builds on the static version by adding a GPS module, enabling georeferenced data collection. It is suitable for heat-related mobile deployments, such as on-foot monitoring (attachable to backpacks, etc.), or installations on bicycles and vehicles. The same Jupyter notebook as above can be used to analyze the collected data.
• **Extended Use Case (3)**: In addition to all components of the mobile standard set-up, this use case integrates a particulate matter sensor. It is tailored for air quality monitoring applications that require the measurement of PM1.0, PM2.5, PM4 and PM10. The Jupyter notebook for this use case is available in the source file repository.
• **Vision Use Case (4)**: This use case uses the same components of the mobile standard setup, but replaces the Arduino Nano 33 BLE Sense [8]with an Arduino Nicla Vision board [9]. It enables camera-based sensing to run visual AI models such as image detection and classification. Unlike the previous use cases, this setup does *not* include a ready-made Jupyter notebook, as it uses a more advanced vision-based board intended for custom applications. Users are encouraged to define their own sensing logic based on specific project needs.

The same base enclosure and PCB are used in all use cases, with extra 3D printed parts added as needed, for example, to support additional sensors in the extended setup (see Fig. 2). This reinforces Octopus’s modularity and ease of customization.

## 2 Hardware description

Octopus consists of a custom oval-shaped PCB (see Fig. 10) and a 3D printed enclosure. The PCB is a 4-layer board (approx. 70 × 50 mm) that integrates a Li-ion battery charger, RGB LEDs on its upper and lower surfaces, a micro-SD card socket, a user button, multiple connector headers, and various discrete electronic components. Two rows of sockets mate with Arduino-standard pins: this allows either an Arduino Nano 33 BLE Sense Rev 2 [8] or an Arduino Nicla Vision board [9] to be plugged in. The PCB also provides Qwiic-compatible [10] connectors for up to four I${}^{2}$C sensor modules: e.g. a Sensirion SPS30 particulate matter (PM) sensor [11], a U-blox GPS module [12], a fan, and I${}^{2}$C breakout pins. The design files for the PCB (Gerber and schematic) are available in the hardware repository.

The electronics are arranged to minimize size and power draw. The Arduino-family board runs on 3.3 V and handles sensor interfacing (via I${}^{2}$C, SPI, UART, etc.) and data logging. Onboard charging allows the 3.7 V Li-ion battery to be recharged via USB. The 500 mAh battery provides approximately 2 h of operation with all sensors active. Expected battery life varies significantly depending on the sampling interval and the active sensors. Power-sensitive applications can leverage the Nano 33’s low sleep currents for long-term monitoring. All firmware and libraries are open-source (see the Octopus library repository).

The Octopus enclosure allows the PCB to slide and lock into place. It can be printed in PLA or PETG materials and includes mounts and slots to hold the board and sensors. Instead of screws, our design uses elastic bands and 3D-printed, built-in threads to secure components, simplifying assembly. Overall, the hardware is highly modular: different Arduino boards and breakout sensors can be attached; unused headers can be left empty. This flexibility supports a wide range of use cases-for example, a temperature logger, an air-quality monitor, or an edge visual AI prototype, without requiring changes to the core system. These capabilities reflect the core design goals of Octopus: low cost, modularity, ease of fabrication, and the ability to be deployed anywhere.

## 3 Design files summary

**Table:** 

Design filename	File type	Open source license	Location of the file

Hardware	Gerber Files	CC BY 4.0	https://doi.org/10.17632/jxjfd7k8z4.2
Firmware	.ino, .cpp, .h files	CC BY 4.0	https://doi.org/10.17632/jxjfd7k8z4.2
Enclosure	STL Files	CC BY 4.0	https://doi.org/10.17632/jxjfd7k8z4.2
BOM	CSV File	CC BY 4.0	https://doi.org/10.17632/jxjfd7k8z4.2
Enclosure	CAD files	CC BY 4.0	https://doi.org/10.17632/jxjfd7k8z4.2
Software	Jupyter Notebook	CC BY 4.0	https://doi.org/10.17632/jxjfd7k8z4.2

			

## 4 Bill of materials summary

An overview of all hardware components that are compatible with Octopus can be found in Table 1, while components for the enclosure are in Table 2.

* These components were bought in sets, lowering their individual cost.

**Table 1:** Hardware components.

Designator	Component	Number	Cost per unit (USD)	Total cost (USD)	Source of materials	Material type	Use Case
Arduino Nano 33 BLE Sense Rev 2*.*	Microcontroller	1	25	25	Digikey	Electronics	1,2,3
Arduino Nicla Vision	Microcontroller	1	115	115	Digikey	Electronics	4
SPS30	PM2.5 particle sensor	1	36.63	36.63	Digikey	Electronics	3
GPS	Sparkfun GPS breakout - chip ant	1	22.02	22.02	Digikey	Electronics	2,3
Lithium Battery	Lithium ion battery - 500mAh	1	7.98	7.98	Sparkfun electronics	Electronics	1,2,3,4
SD Card	MicroSDHC card	1	7.33	7.33	Amazon	Electronics	1,2,3,4
Fan	5V DC fan 40 × 40 mm	1	0.48	0.48	Winsinn	Electronics	1,2,3,4
PCB	Costum PCB	1	13.12**	13.12**	Table 3	Electronics	1,2,3,4

**Hardware use case 1**			**53.91**	**53.91**			
**Hardware use case 2**			**75.93**	**75.93**			
**Hardware use case 3**			**112.56**	**112.56**			
**Hardware use case 4**			**143.91**	**143.91**

**Table 2:** Enclosure components.

Designator	Component	Number	Cost per unit (USD)	Total cost (USD)	Source of materials	Material type
3D printing material	220 g PLA Basic filament	1	4.78*	4.78*	Bambulab	Other
Mini rubber bands	Mini rubber bands	2	0.05*	0.05*	Amazon	Other
1/4” inch insert	Tripod insert	1	0.47*	0.47*	Amazon	Other

Enclosure components			**5.3**	**5.35**		

* These components were bought in sets, lowering their individual cost.

** The bill of materials for the Octopus PCB is reported in Table 3.

** The bill of materials for the Octopus PCB is reported in Table 3.

**Note:** In order to attach the enclosure to other surfaces, it is necessary to purchase additional components. Potential attachments consist of a wide range of possibilities, including, but not limited rubber bands, magnets, or strands. The enclosure includes a standard 1/4 inch insert compatible with standard photography tripods.

**Table 3:** Printed circuit board.

Designator	Component	Qty/unit	Batch of 1 (USD)	Batch of 1000 (USD)	Source	Material
PCB	CS1	1	8	–	https://pcbway.com	PCB
C1, C6, C8, C9, C11	CC0805KRX7R9BB104	5	0.08	13.60	https://digikey.com	Electronics
C2,C3	C2012X5R1H475K125AB	2	0.47	140.20	https://digikey.com	Electronics
C4,C5	CL21A106KAYNNNE	2	0.15	34.54	https://digikey.com	Electronics
C7	CB5R5334HF-ZJP	1	1.45	563.70	https://digikey.com	Electronics
C10	CL21A226MPQNNNE	1	0.15	34.46	https://digikey.com	Electronics
DS1, DS2, DS3, DS4, DS5	PMEG1030EJ	5	0.27	105.72	https://digikey.com	Electronics
J1	B2B-PH-K-S	1	0.12	73.95	https://digikey.com	Electronics
J4	SSM-102-L-SV	1	0.71	434.88	https://digikey.com	Electronics
J5	SSM-108-L-SV	1	1.92	1,209.38	https://digikey.com	Electronics
J6	SSM-109-L-SV	1	2.11	1,343.05	https://digikey.com	Electronics
J7	BM04B-SRSS-TBT	1	0.71	460.02	https://digikey.com	Electronics
J8	B5B-ZR-SM4-TF	1	0.64	409.88	https://digikey.com	Electronics
J9	MSD-4-A	1	0.36	259.38	https://digikey.com	Electronics
J10	S2B-XH-A	1	0.12	73.60	https://digikey.com	Electronics
LD1	150141YS73100	1	0.19	140.76	https://digikey.com	Electronics
LD2, LD3	T3A33BRG-H9C0002X1U1930	2	0.27	238.07	https://digikey.com	Electronics
L1	LQM2HPN4R7MG0L	1	0.17	94.14	https://digikey.com	Electronics
PB1	FSM4JSMAATR	1	0.34	216.16	https://digikey.com	Electronics
R1	RC0805FR-07120KL	1	0.10	12.79	https://digikey.com	Electronics
R2, R5	RC0805FR-07120KL	2	0.10	7.14	https://digikey.com	Electronics
R3	CRCW0805430KFKEA	1	1.10	12.79	https://digikey.com	Electronics
R4	RC0805FR-072K8L	1	0.10	8.16	https://digikey.com	Electronics
R6	RC0805FR-07180KL	1	0.10	8.16	https://digikey.com	Electronics
R7, R8	CRCW08054K70FKEAC	2	0.10	10.59	https://digikey.com	Electronics
R9, R10, R11, R12, R13, R15, R16	RMCF0805FT33K0	7	0.10	6.58	https://digikey.com	Electronics
R14	RC0805FR-07470RL	1	0.10	7.14	https://digikey.com	Electronics
R17	RMCF0805FT10K0	1	0.10	6.58	https://digikey.com	Electronics
R18	311-1.5KARCT-ND	1	0.10	7.14	https://digikey.com	Electronics
TVS1	DT2042-04SO-7	1	0.39	120.00	https://digikey.com	Electronics
U1	MCP73833T-AMI/UN	1	1.14	880.00	https://digikey.com	Electronics
U2	MCP1642B-50I/MS	1	1.12	850.00	https://digikey.com	Electronics
U3	RX-8025SA:AA	1	9.75	3,849.68	https://digikey.com	Electronics

PCB components			**40.53**	**13.12**		

**Note:** In order to attach the enclosure to other surfaces, it is necessary to purchase additional components. Potential attachments consist of a wide range of possibilities, including, but not limited rubber bands, magnets, or strands. The enclosure includes a standard 1/4 inch insert compatible with standard photography tripods.

The total cost of Octopus, depending on the chosen use case, can be found in Table 4. The total price is calculated by adding the cost of hardware materials (Table 1) and enclosure components (Table 2).

**Table 4:** Total cost per use case.

Number	Use case	Total cost
1	Static standard use case	59.26
2	Mobile standard use case	81.28
3	Extended use case	127.91
4	Vision use case	149.26

## 5 Build instructions

The Octopus environmental sensing platform is designed for fast, user-friendly assembly, typically taking about 20 min to set up with basic electronics workshop tools and access to a 3D printer. To accommodate a variety of monitoring scenarios, Octopus supports four modular configurations, ranging from basic temperature and humidity logging to more advanced configurations with GPS, air quality sensors, or visual monitoring capabilities. Each configuration builds on a core hardware base, allowing users to scale the system depending on their data collection goals and environmental conditions (e.g., mobile use, high-heat climates, particulate measurement, or visual sensing). A cooling fan is required for hotter environments.

### 5.1 Octopus PCB and electronics

The PCB can be ordered fully assembled from a PCB fabrication service using the Gerber files and the BOM available in the source file repository. The 4-layer design was chosen for signal integrity and noise reduction. Environmental sensors, particularly for humidity and particulate matter, are sensitive to electrical noise. The additional layers provide dedicated ground and power planes that improve signal quality and reduce electromagnetic interference between components. While 4-layer boards have slightly higher per-unit cost, the difference is minimal at typical PCB fabrication services (roughly 1-2 USD more per board), and the improved reliability reduces troubleshooting time for novice users. The service will source and solder the components listed in the bill of materials. The Octopus PCB’s subsystems are summarized in the schematics reported in Fig. 5. Detailed descriptions of each subsystem follow below.

**Fig. 3:**
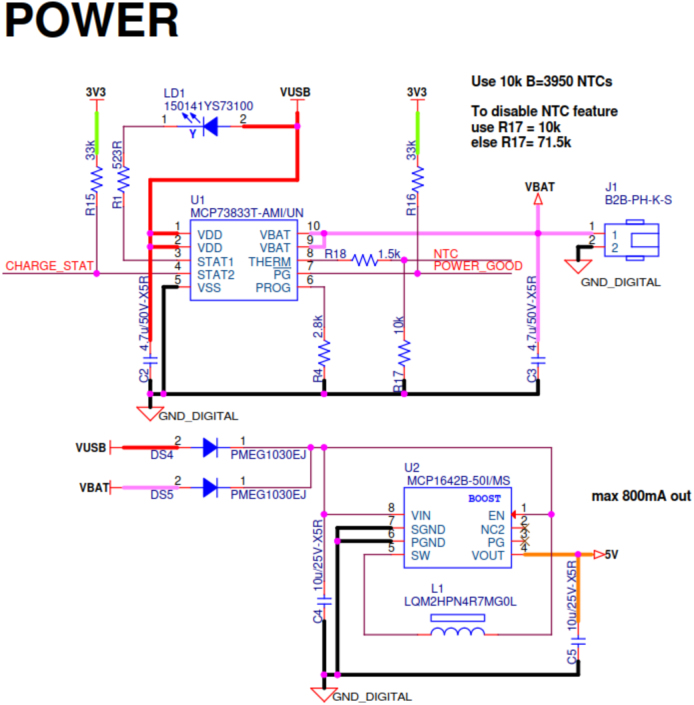
Power regulation circuit with MCP73833 charger and boost converter.

**Fig. 4:**
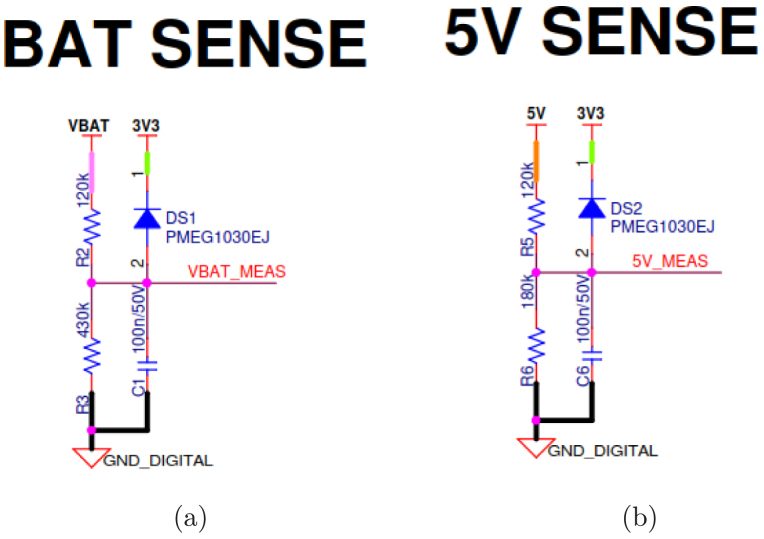
Battery and voltage sensing divider. (a) battery sensing, (b) 5V USB voltage sensing.

**Fig. 5:**
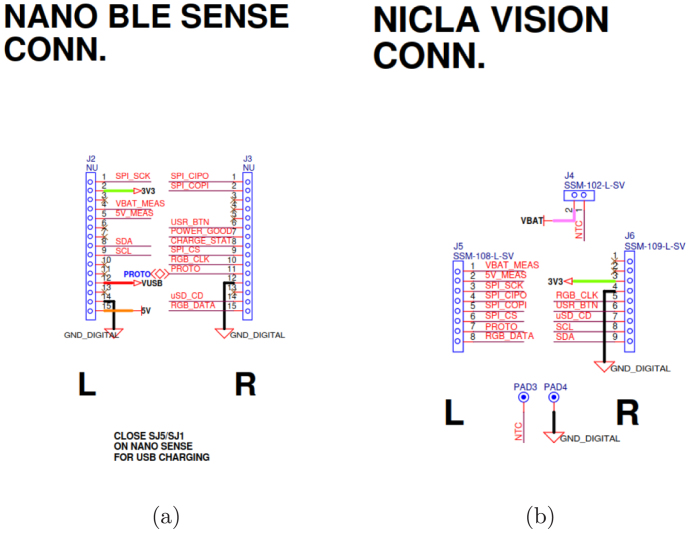
Microcontroller interfaces: (a) Arduino Nano, (b) Arduino Nicla Vision.

**Fig. 6:**
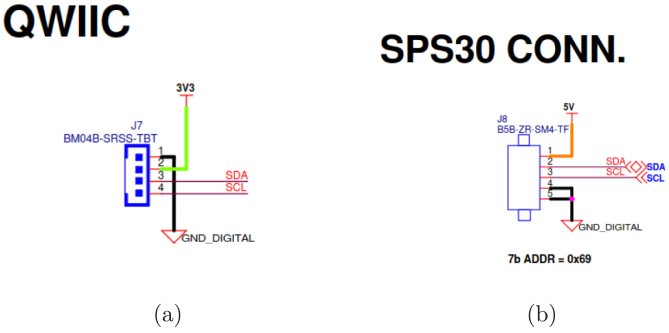
I${}^{2}$C Sensors and indicators: (a) Qwiic, (b) SPS30.

**Fig. 7:**
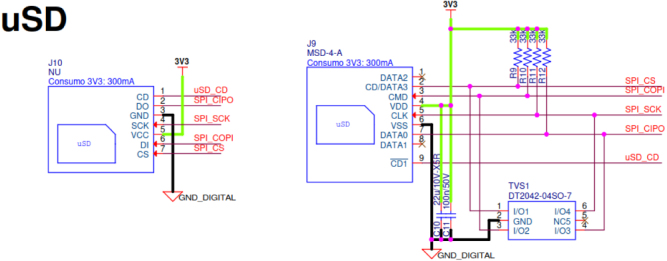
Storage and user input.

• **Power Regulation and Charging**, Fig. 3: The Power is managed by a linear Li-ion charger and boost converter. The MCP73833 linear charger (U1) accepts USB VBUS (5V) and charges the single-cell Li-ion battery (VBAT) at about 500 mA. Its charge output provides a charge-status indication (LD1). The battery voltage VBAT can drive a boost regulator (MCP1642B, U2) to produce a regulated 5V output for peripherals (up to 800 mA, see Fig. 3 . Schottky diodes on the VUSB and VBAT lines prevent reverse current flow when only one source is present. A 3.3V LDO (on-board) derives the logic supply from the battery for the digital circuitry as needed. These power management components collectively ensure safety for novice users: the JST-PH connector prevents reverse polarity insertion; the Schottky diodes prevent reverse current flow; the MCP73833 includes built-in thermal protection; the boost converter provides current limiting(800 mA max); and the TVS diode offers ESD protection. The enclosure design physically protects electronics from accidental contact.
• **Voltage Sensing**, Fig. 4: Both the battery and the USB input voltages are monitored by the microcontroller’s ADC. The VBAT divider Fig. 4(a) uses R2 to scale the battery voltage into the 0–3.3V range; a Schottky diode (DS1, PMEG1030EJ) clamps the VBAT MEAS pin to 3.3V for protection. Likewise, the 5V USB input is sensed by (R5 120 kilo-ohm) and (R6 180 kilo-ohm), feeding the 5V MEAS pin in Fig. 4(b), with clamp diode DS2. These high-impedance dividers allow firmware to continuously measure the battery level and detect the presence of external 5V power without loading the sources.
• **Microcontroller Interface**, Fig. 5: The board is designed to host either an Arduino Nano 33 BLE Sense or an Arduino Nicla Vision module, using dedicated connectors that break out their pinouts.
• **I**${}^{2}$**C Sensors and Indicators** A Qwiic-compatible I${}^{2}$C header (J7) provides 3.3V, GND, SDA, and SCL for plugging in I${}^{2}$C sensors. The Sensirion SPS30 particulate-matter sensor connects via a five-pin header Fig. 6(a) powered by 5V; it communicates on I${}^{2}$C (default address 0x69). Also on the I${}^{2}$C bus is a supercapacitor-backed real-time clock (RX-8025SA, 5.5μ A typical). The RTC automatically switches to the backup supercapacitor when the main 3.3 V is off, and it appears on I${}^{2}$C at address 0x32. (Note: the Nano module provides the SDA/SCL pull-ups for the bus.) Two addressable RGB LEDs serve as visual indicators. Both LEDs share the 5 V supply and ground. The microcontroller drives a common clock line (RGB CLK) and sends serial color/data words (RGB DATA) into the bottom LED. In this way firmware can set colors or animations for status indication. For users planning to integrate non-Qwiic sensors, the PCB includes I2C breakout pins in addition to the Qwiic connectors. The Arduino pin headers are also accessible for connecting sensors using SPI, UART, or analog interfaces.
• **Storage and User Input**: For data logging, the board provides a microSD card socket. The SD card’s SPI lines (CLK, CMD, D0–D3) are routed through a TXS0108E 8-bit level shifter (U3). A user pushbutton (PB1) is also provided. This switch connects the USR BTN to ground when pressed. A 30 kohms pull-up resistor (R13) holds the line at 3.3 V when the button is released. The MCU can read this GPIO (active low) to implement simple user controls (e.g. start/stop logging). (see Fig. 7)
• **Electromagnetic Interference Considerations**: Electromagnetic interference (EMI) influenced the component placement design. The GPS module is positioned in the bottom enclosure section, physically separated from the main PCB. The SPS30 particulate-matter sensor uses I2C communication, which is relatively robust against interference, and its placement in the middle enclosure section provides additional separation. The fan operates at 5 V DC and does not produce significant RF interference. The 4-layer PCB design with dedicated ground planes helps contain noise from switching components.

### 5.2 Octopus assembly

This section provides configuration-specific assembly instructions for Octopus, corresponding to the four main use cases defined in Section 1.2. The enclosure design files were tested across multiple 3D printers including Bambu Lab and Ultimaker printers. Print quality and functionality are consistently preserved across different printers. Minor variations in printer-specific tolerances can slightly affect the fit of the embedded screw-style attachment mechanism, but the design is tolerant enough that the mechanism functions reliably across all tested printers. All configurations share a common PCB and enclosure, with added components depending on the specific application.

#### Static standard use case (1).

**Fig. 8:**
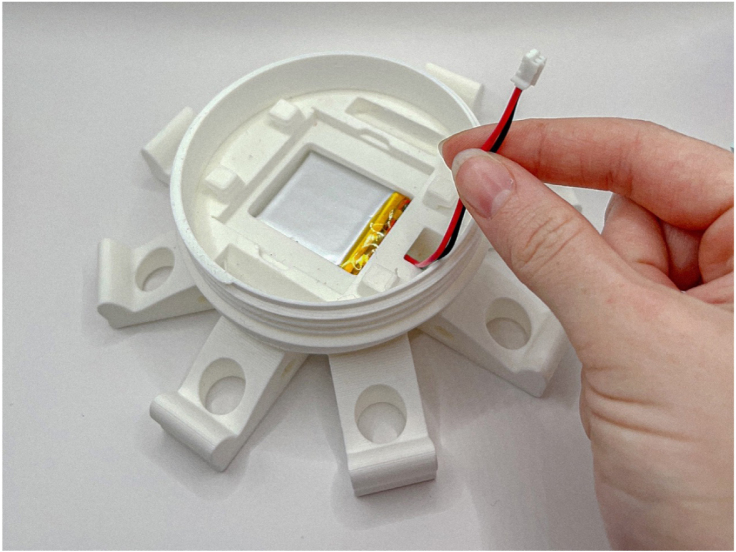
Prepare bottom layer: insert battery.

**Fig. 9:**
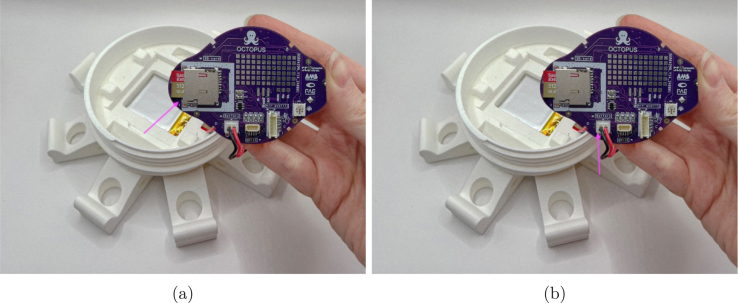
PCB placement in top layer: (a) insert SD card, (b) plug battery.

**Fig. 10:**
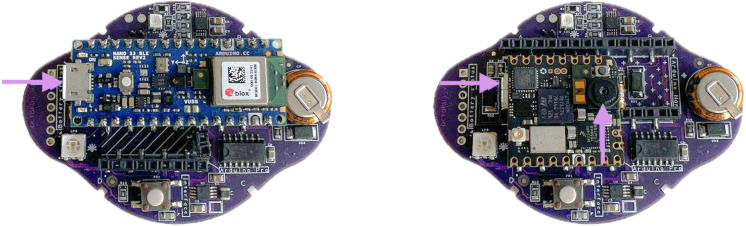
Assembly direction of micro-controllers.

**Fig. 11:**
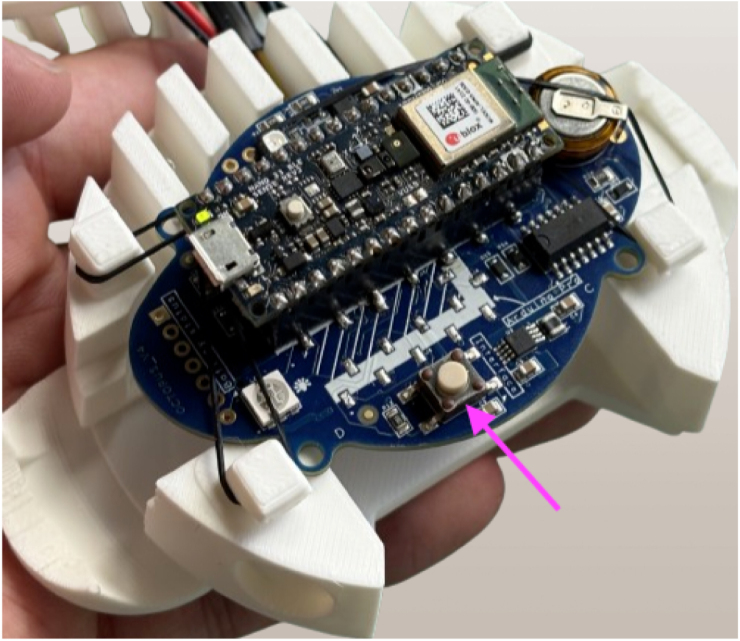
PCB placement.

**Fig. 12:**
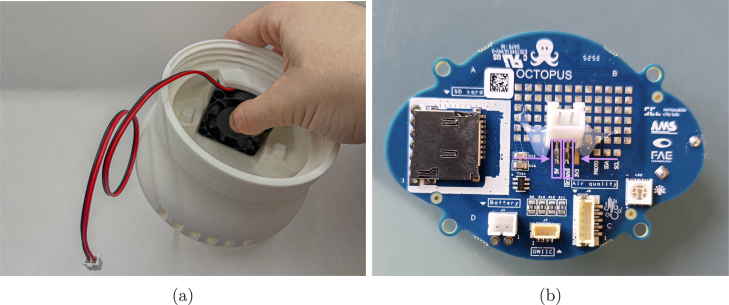
Fan placement: (a) fan placement, (b) soldering.

1. Prepare the Bottom Layer, Fig. 8: Place the Li-Po battery into the bottom section of the 3D printed enclosure, which serves as the base of the device. The battery is secured in place using elastic bands wrapped around the enclosure’s built-in posts, ensuring the component is firmly held against the housing to prevent movement or vibration during deployment.
2. Prepare the Top Layer, Fig. 9: Insert a microSD card into the slot provided on the custom printed circuit board. Connect the battery’s plug to the PCB at the header labeled battery. Mount the Arduino Nano 33 BLE Sense onto the PCB. Ensure correct orientation: the USB Type-C connector should point in the opposite direction from the real-time clock (RTC) module (Fig. 10).
3. Insert the PCB into the top 3D printed enclosure (Fig. 11). Press the user push button (pink arrow) to turn on the device, and secure the PCB with elastic bands around the enclosure pegs.
4. All deployments must include the cooling fan to maintain proper airflow where ambient temperatures are high or the device is exposed to direct sunlight. Snap into place and connect to 5 V/GND on PCB (Fig. 12).
5. Assemble the top and bottom enclosure sections.

#### Mobile standard use case (2).

This version builds on the static standard use case by adding a GPS module.

**Fig. 13:**
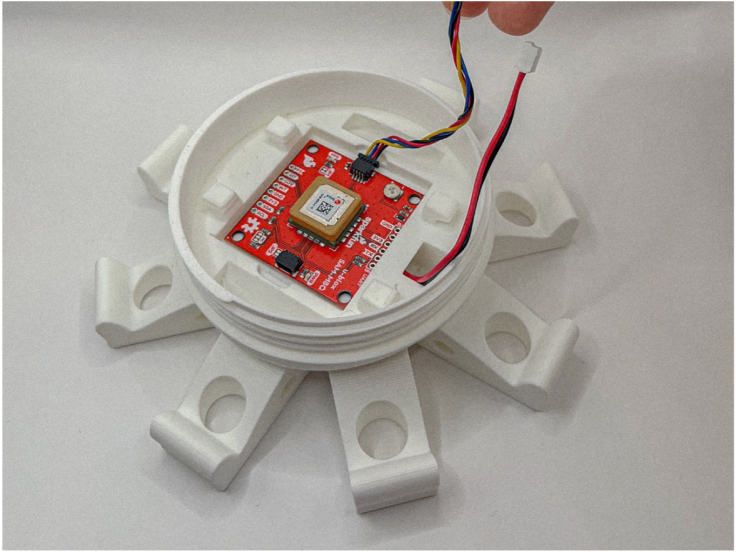
Insert GPS.

**Fig. 14:**
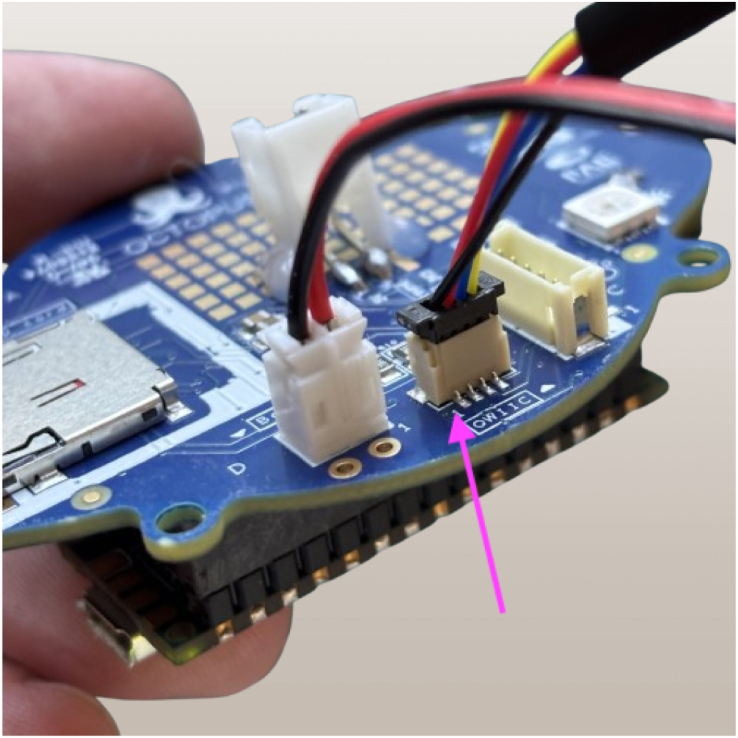
Connect Qwiic to PCB.

**Fig. 15:**
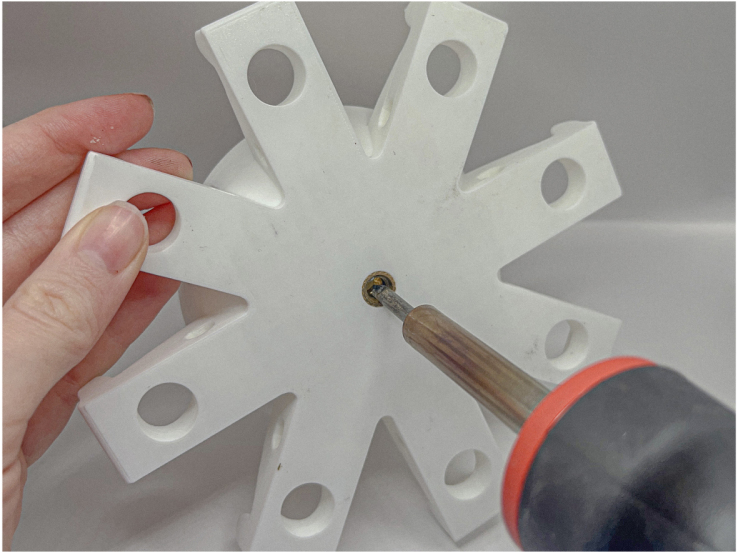
Tripod mount.

1. Place the GPS module on top of the battery in its designated slot in the bottom enclosure (Fig. 13). Secure with elastic bands.
2. Connect the GPS module to the PCB using the Qwiic connector (Fig. 14). Ensure that the cables are long enough to avoid pinching.
3. Optional: The tripod and magnet mount are shared among all use cases: Install a 1/4”-20 threaded insert into the base using a soldering iron (Fig. 15).

#### Extended use case (3).

This configuration adds a middle enclosure section to house a SPS30 particulate matter sensor.

**Fig. 16:**
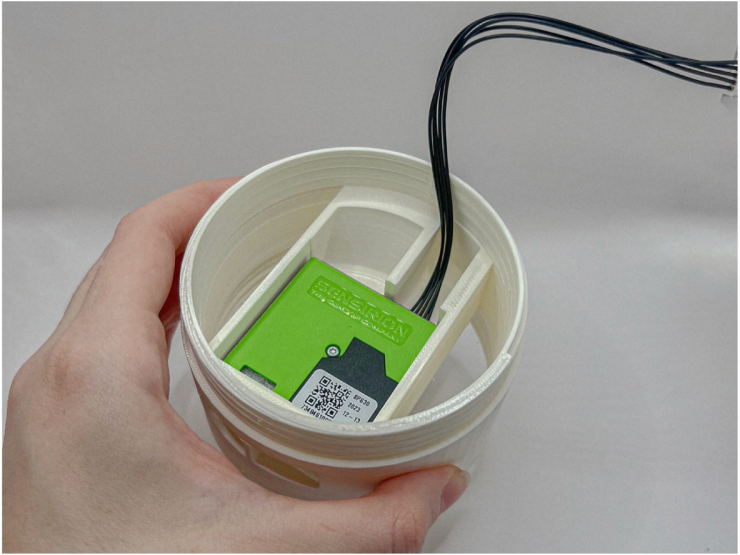
SPS30 placement in middle section.

**Fig. 17:**
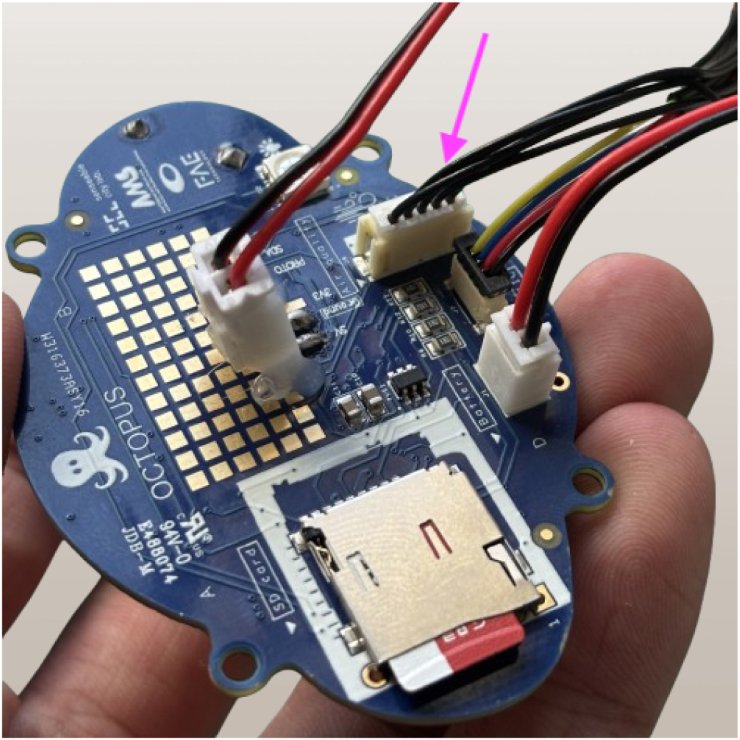
Plug SPS30 sensor to PCB.

**Fig. 18:**
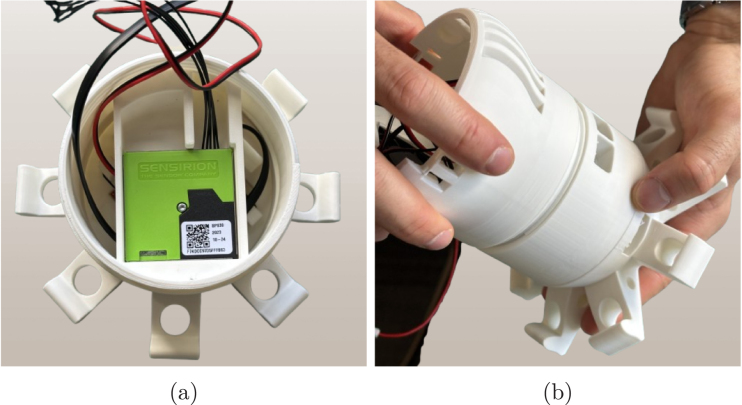
Assemble full device: (a) attach middle section, (b) assemble device.

1. Insert the SPS30 into the middle enclosure with proper alignment (Fig. 16). Connect the sensor to the PCB (Fig. 17).
2. Stack and screw together the bottom, middle, and top enclosure sections. Ensure all cables are properly routed and are long enough to accommodate for the twisting that happens when closing the device (Fig. 18).

#### Vision use case (4).

This configuration swaps the Nano 33 BLE Sense with the Nicla Vision for image-based applications.

1. Follow use case 2 steps for battery, SD card, GPS module, and enclosure assembly.
2. Mount the Nicla Vision board in the proper orientation with the camera facing up (Fig. 10-right).
3. Press the onboard power button to turn on the device (Fig. 11).

## 6 Operation instructions

### 6.1 Octopus library setup

The Arduino Integrated Development Environment (IDE) [13] is used for programming the Octopus. All necessary firmware functions are provided through the custom Octopus Arduino library, which is available via the Arduino library manager [14] (see Table 5).

**Table 5:** Octopus IDE setup steps.

Step	Action
1	Install Arduino IDE on the development computer.
2	In the Library Manager, search for “Octopus” and install the library published by MIT Senseable City Lab.
3	When prompted, approve and install all dependency libraries.
4	In Boards Manager, install Arduino Mbed OS Nano Boards (for Arduino Nano 33 BLE Sense).
5	Still in Boards Manager, install Arduino Mbed OS Nicla Boards (for Arduino Nicla Vision).
6	Connect the Arduino to the computer via USB, then in Tools select the correct Board (e.g., “Arduino Nano 33 BLE”) and the Port (COM/tty).

### 6.2 Firmware development and upload

The Octopus library includes example sketches that serve as reference firmware. A ready-to-use sample named octopus-sample for a standard use cases can be accessed from the Arduino IDE’s example menu. This example comes pre-configured to initialize the Octopus hardware and log sensor data, making it an ideal starting point for testing.

The IDE will compile the firmware and flash it to the Octopus microcontroller over the USB connection. During the upload process, the board’s built-in LED will typically blink to indicate bootloader activity (for instance, an orange LED on the Nano 33 BLE will pulse while waiting for the upload). After a few seconds, the firmware is done uploading and flashed, Octopus will reboot and start running the new firmware.

If successful, the Octopus’s status LED will emit a steady purple light on the RGB LED to confirm the firmware is running correctly (see Table 6).

If the firmware does not initiate properly, the user can press the reset button on the microcontroller. Once the device indicates a successful start, the Octopus’s software setup is complete.

**Table 6:** Octopus RGB LED status codes.

LED color/pattern	Meaning
Steady Purple	Firmware running correctly
Steady Blue	Cold temperature detected
Blinking Red	Error state or sensor initialization failed

### 6.3 System operation

• With the Octopus sample firmware installed, the Octopus platform operates autonomously to collect environmental data. On power-up, the firmware performs an initialization routine for all sensors and subsystems. The Octopus Arduino library is used to configure the onboard sensors (e.g., temperature, humidity sensors on the Nano 33 BLE) as well as any attached external sensors, such as the GPS module and the SPS30 particulate matter sensor.
• For each sensor part, the firmware checks for successful startup and prints an initialization status message via the serial console for debugging purposes (e.g. “GPS initialized”.). After sensor initialization, the device’s real-time clock and SD card interface are started. The firmware performs sequential sensor initialization, checking each sensor independently. If a sensor fails to initialize (e.g., not connected or communication error), the firmware logs the failure but continues initializing remaining sensors, allowing partial operation rather than complete failure. Users can check the serial output to identify which sensor failed. Data logging proceeds with available sensors, marking unavailable data fields as ‘na’ in the CSV output. The firmware mounts the microSD card and prepares a new data file for logging in CSV format. Each record follows a consistent schema, depending on the use case. Once setup is complete, the microcontroller enters the main data acquisition loop. The firmware writes data to the SD card in real-time using appended operations to CSV files. This approach means that data up to the most recent write will be preserved if power is lost.
• During operation, Octopus continuously reads data from all available sensors at a user-set interval (for example, every 5 s in the default firmware configuration). At each interval, the system obtains a timestamp from the configured source; this can be modified via the Octopus library to prioritize GPS or RTC, reads the available sensor values (such as air temperature, relative humidity, and PM2.5), and, if available, reads the current location from the GPS. These readings are formatted into a single comma-separated record and written to the microSD card in CSV format. The specific data schema depends on the selected use case. For the static standard use case, the fields include Timestamp, Temperature, Humidity. The mobile standard use case adds geolocation, resulting in Timestamp, Latitude, Longitude, Temperature, Humidity. The extended use case further includes air quality data as Timestamp, Latitude, Longitude, Temperature, Humidity, PM1.0, PM2.5, PM4, PM10.0. Finally, the vision Use Case supports application-specific outputs, such as Timestamp, Image Filename, or other vision-derived data, depending on user-defined logic. The firmware appends each record to the data log on the SD card in real time using the Octopus library’s logging function. In parallel, the data can also be output to the serial port for live monitoring: the firmware uses Serial.println() to print each sensor reading and the data line to the serial interface. This allows users to observe sensor values in real time using a computer during testing or debugging (e.g., via the Arduino IDE’s Serial Monitor).
• The Octopus device provides user feedback through an integrated RGB LED on the PCB. The firmware uses the onboard LED to visually communicate system status through predefined colors and blink patterns. Some behaviors are hardcoded in the firmware; for example, a steady purple light indicates successful initialization, while a blinking red light means a system error or sensor failure. Other patterns may be defined by the firmware library or configured by the user for specific application needs.
• Common issues include SD card initialization failures (formatting or card compatibility), sensor non-detection (I2C address conflicts or wiring errors), battery charging failures (USB connection or charge LED indicators), and firmware upload failures (bootloader mode or port selection). Serial console output during system initialization provides diagnostic messages for each subsystem to aid fault identification.
• To avoid errors arising from the use of low-cost sensors, it is recommended that:(1) pre-deployment calibration against a reference device allows users to characterize individual sensor biases; (2) offset corrections can be implemented in firmware or post-processing using calibration coefficients; (3) the enclosure design with fan-assisted ventilation reduces self-heating errors common in enclosed sensors.

### 6.4 Data retrieval

Power off the device, press and hold the power button for 5 s until the top RGB LED indicator turns off. Then remove the microSD card from its slot. The microSD card is then connected to a computer using a card reader. The logged data file(s) can be copied to the computer for analysis. At this stage, the data is still raw and unprocessed; the Octopus firmware does not perform any data processing or analysis on board to conserve microcontroller resources.

### 6.5 Data analysis

After retrieving the raw data from the microSD cards, Jupyter notebooks can be used for further data analysis. This will allow the environmental data collected by the Octopus platform to be explored, analyzed, and displayed. Two Jupyter Notebooks are currently available for use (see the repository).

The first is intended for static deployments (Standard use case (1)), and the second for mobile deployments (Mobile standard use case (2) and Extended use case (3)). The initial section of the notebooks will demonstrate how to import the data and libraries, followed by a series of data pre-processing steps, which include handling missing values, outlier detection and removal, resampling, normalization, and smoothing. These steps are crucial when dealing with raw data in order to limit data points that might skew the results. Furthermore, the notebook provides examples on how to plot time series graphs for all use cases.

The second notebook, meant to support use case 3, delves more deeply into the data analysis process as it includes processing of GPS coordinates, thus enabling the execution of mapping and clustering analysis. The examples employ the folium library [15] to create an interactive map for the plotted values. Hierarchical clustering through DBSCAN [16] is performed to analyze hotspots in the collected datasets.

## 7 Validation and characterization

In this paper, we introduced Octopus, a low-cost, modular, and open-source environmental sensing platform tailored for makers. Designed for flexibility and ease of use, Octopus empowers individuals to participate in environmental data collection across urban and ecological contexts. By open-sourcing all hardware, software, and mechanical components, alongside extensive documentation and example use cases, the platform helps to lower the barrier to entry for environmental data collection for users with varying levels of technical expertise.

### 7.1 Field performance comparison with reference device

**Fig. 19:**
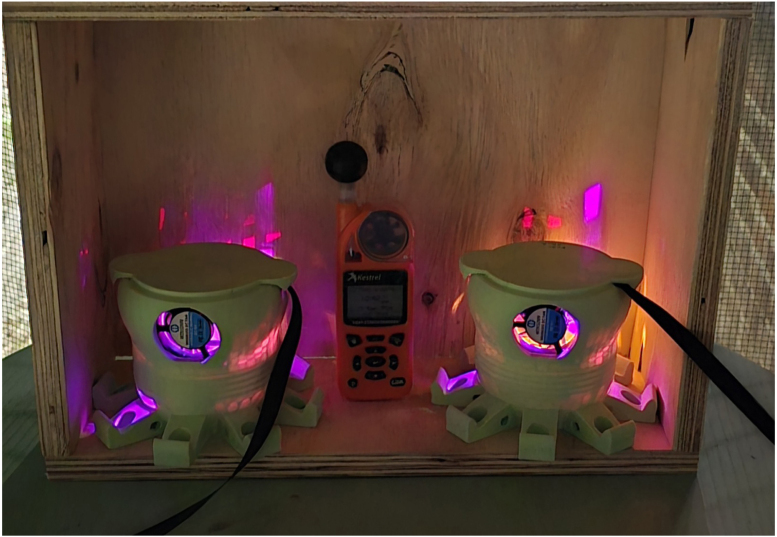
Calibration setup: (left) Octopus sensor 1, (center) Kestrel reference, (right) Octopus sensor 2.

We calibrated and evaluated the Octopus (static standard case) using collocated measurements against a reference instrument for application in the ambient outdoor environments. This study focused on validating the standard use case of Octopus and did not include other use cases, such as the extended use case with the particulate matter sensor. Although the US EPA (United States Environmental Protection Agency) does not explicitly recommend a testing protocol for low-cost temperature and humidity sensors, the experimental setup was based on the US EPA’s recommendation to test the performance evaluation of low-cost air quality sensors [17]. We calibrated two Octopus (Static standard case) against a reference meteorological monitoring device manufactured by Kestrel (model 5400, hereafter referred as Kestrel). The Kestrel 5400 is a well-established, multi-parameter environmental meter that is individually tested to NIST-traceable standards, with manufacturer-stated accuracies of ±0.5 °C for temperature and ±2% RH for humidity. Using only one reference could limit cross-checking; this was due to the logical constraint, as only one device was available during the field tests. Future validation efforts are planned to involve multiple reference instruments or laboratory calibrations for redundancy and traceability. In particular, we intend to expand inter-sensor validation by comparing Octopus readings against additional reference devices (or multiple Kestrel units) and possibly against controlled environmental chamber data. The two octopus devices were operated next to Kestrel in the ambient outdoor environment over a continuous period of approximately 18 h (Fig. 19). The field validation was conducted over an approximately 18-hour period due to practical deployment constraints and study design considerations. This deployment spanned an overnight cycle, from late afternoon through the following morning, allowing the sensors to experience both daytime heating and nighttime cooling conditions. As a result, the validation captured a substantial range of temperature and relative humidity, including key phases of the diurnal cycle such as evening cooling, nighttime stabilization, and morning warming. This duration was sufficient to observe clear diurnal variability and to evaluate sensor response, potential lag, and bias under dynamically changing environmental conditions. Future validation efforts will extend to multi-day deployments to assess long-term stability and performance across full diurnal cycles and a wider range of meteorological conditions. Before collecting the data we made sure that the clocks for all the devices were synchronized. For the Octopus devices, this was accomplished by setting the onboard real-time clock (RX-8025SA) during the setup phase. The logging start was coordinated manually with all devices powered on within the same minute. This procedure provides sufficient synchronization for environmental monitoring where conditions change on timescales of minutes to hours. Data were logged every 5 s and subsequently aggregated to one-minute averages for analysis. Outliers were identified and removed to improve data quality. Throughout the measurement period, the devices were properly ventilated and shielded from direct solar radiation to minimize environmental biases, while continuously capturing ambient temperature and humidity data. This setup allowed for a controlled yet fully outdoor evaluation of sensor performance under real-world conditions, providing robust data for regression analysis and sensor comparison.

**Fig. 20:**
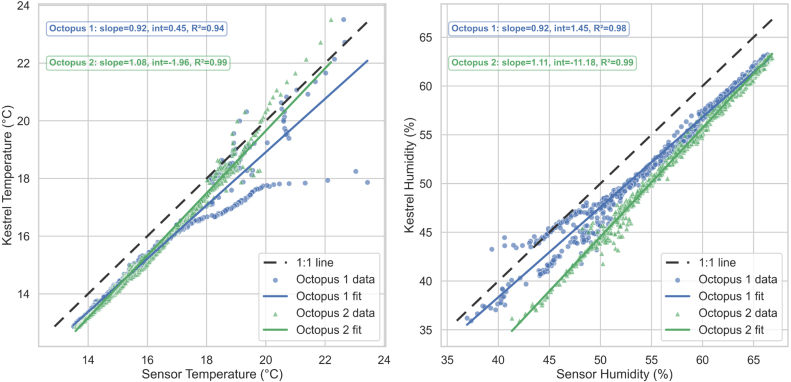
Regression plot of the two Octopus sensors against the reference instrument Kestrel.

To evaluate the performance of the Octopus sensors, we applied linear regression analysis, with the Octopus sensor measurements placed on the x-axis (independent variable) and the reference Kestrel measurements on the y-axis (dependent variable). Several regression parameters were used to characterize performance. The slope indicates the sensitivity of the sensor; a slope of 1 represents perfect agreement with the reference, while slopes less than 1 indicate underestimation and slopes greater than 1 indicate overestimation. The coefficient of determination (R${}^{2}$) reflects how much of the variation in the reference measurements can be explained by the sensor, with values close to 1 showing strong agreement in temporal variability. The mean bias represents the average offset between sensor and reference, where smaller values indicate higher accuracy. The root mean square error (RMSE) quantifies the overall deviation, capturing both systematic and random errors. Finally, the coefficient of variation (CV) provides a normalized measure of variability, allowing inter-sensor precision to be compared; lower CV values indicate stronger agreement. The regression analysis demonstrates that the Octopus sensors are capable of closely tracking the reference Kestrel measurements for outdoor environmental monitoring (Fig. 20). For temperature, Octopus 1 exhibited a slope of 0.92 and Octopus 2 a slope of 1.08, with R${}^{2}$ values of 0.94 and 0.99, respectively. Both slopes are close to unity, demonstrating that the sensors captured the thermal variability with good precision, although Octopus 1 showed a slight underestimation and Octopus 2 a slight overestimation relative to the Kestrel. The biases were modest (0.80 °C for Octopus 1 and 0.65 °C for Octopus 2), and the RMSE between the two Octopus devices was only 0.59 °C with a CV of 3.51%, indicating reasonably good inter-sensor precision.

For relative humidity, Octopus 1 showed a slope of 0.92 and Octopus 2 a slope of 1.11, with R${}^{2}$ values of 0.98 and 0.99. The slopes again indicate small differences for both devices, relative to the Kestrel. The biases were 2.89% for Octopus 1 and 4.60% for Octopus 2, both within acceptable ranges for low-cost sensors. Inter-sensor agreement was similarly strong, with an RMSE of 2.52% and a CV of 3.44%. Relative humidity values ranged from 36.9% to 66.8%, with a mean of 56.7% compared to a Kestrel mean of 52.8%. These results demonstrate that both Octopus sensors closely captured the magnitude and temporal variability of outdoor humidity. The reference measurements from Kestrel have a manufacturer-specified accuracy of ±0.5 °C for temperature and ±2% RH for relative humidity within its normal operating range. Observed Octopus biases of 0̃.6–0.8 °C and 3̃%–4% RH are therefore comparable to the uncertainty of the reference instrument itself, indicating that Octopus performance approaches the accuracy limits of the reference device. The small bias in the measurements can be addressed further to improve the accuracy and reduce the overall discrepancy in the measurements by applying these strategies: (i) Multivariate/offset calibration: A two-point calibration (covering low and high values) for temperature and humidity could be performed so that Octopus readings align more closely with standard instruments across the range of interest. This could involve multivariate calibration if sensor readings are interdependent (e.g., humidity sensor output affected by temperature). (ii) As low-cost sensors in general have a slight temperature-dependent bias. Implementing temperature compensation algorithms (adjusting the humidity reading based on current temperature, for instance) could reduce errors when conditions change. (iii) Further, we plan to add a user-defined digital filtering or averaging of the sensor readings to reduce random noise and huge fluctuations, leading to more stable outputs, and the removal of outliers. We plan that the future evaluations will involve multi-day deployments of Octopus units to observe long-term stability and performance under varying weather (including full 24-hour cycles, and possibly different weeks or seasons). Overall, these results indicate that the low-cost Octopus sensors perform robustly under outdoor conditions, capturing both temperature and humidity trends with minimal variability and strong agreement with the reference instrument, making them highly suitable for broader deployment in urban environmental sensing networks (Fig. 20).

### 7.2 Scope and limitations of octopus

Octopus is designed as a low-cost, open-source sensing platform and is not intended to replace certified, regulatory-grade environmental monitoring instruments. While the system demonstrates strong performance relative to its cost, it does not meet the calibration, long-term stability, and traceability requirements associated with professional or compliance-oriented instruments. Instead, Octopus is best suited for non-regulatory research applications, including dense sensor networks, exploratory studies, prototyping of new sensing concepts, citizen-science deployments, and educational use, where affordability, flexibility, and high spatial coverage are critical. In such contexts, Octopus can provide valuable indicative measurements and reveal spatial patterns or trends, albeit with higher uncertainty than reference-grade devices. To further strengthen the robustness of the results, future work will include validation using a larger number of Octopus units. We are currently assembling additional devices and plan to conduct an expanded intercomparison exercise to better quantify unit-to-unit variability and assess the scalability of the platform. We also did not perform durability tests for heat, extreme humidity, and continuous operations over days, as this was beyond the scope of the study. The current validation focused on sensor accuracy rather than long-term environmental durability. The PLA/PETG enclosure materials are standard for outdoor maker projects and have known limitations: PLA may deform above 50–60 °C, while PETG offers better heat resistance. UV degradation of PLA/PETG over months of sun exposure is a known issue that users should consider for permanent installations.

For applications requiring precise absolute measurements or regulatory compliance, the Octopus modularity allows for implementing strategies such as including more identical sensors for redundancy or plugging in mid- to high-cost sensors using the Qwiic connector or the breakout pads on the Octopus PCB, enabling freedom to implement the most appropriate cost-accuracy trade-off for a given application scenario.

## CRediT authorship contribution statement

**Oluwatobi Oyinlola:** Writing – original draft, Validation, Methodology, Data curation, Conceptualization. **Åse Håtveit:** Writing – review & editing, Methodology, Data curation, Conceptualization. **Chada Elalami:** Writing – review & editing, Methodology, Data curation, Conceptualization. **Simone Mora:** Writing – review & editing, Supervision, Project administration, Methodology, Data curation, Conceptualization. **Ravish Dubey:** Writing – review & editing, Validation, Methodology. **Fábio Duarte:** Writing – review & editing, Supervision, Project administration, Methodology, Funding acquisition, Data curation, Conceptualization. **Jessica Biagioli:** Methodology, Conceptualization. **Matteo Gregis:** Methodology, Conceptualization. **Carlo Ratti:** Supervision, Funding acquisition, Conceptualization.

## Ethics statements

Not relevant.

## Declaration of competing interest

The authors declare that they have no known competing financial interests or personal relationships that could have appeared to influence the work reported in this paper.
